# Laminated Copper Nanocluster Incorporated Antioxidative Paper Device with RGB System-Assisted Signal Improvement

**DOI:** 10.3390/nano8020097

**Published:** 2018-02-09

**Authors:** Chong-You Chen, Chia-Lin Chen, Chang-Ming Wang, Wei-Ssu Liao

**Affiliations:** Department of Chemistry, National Taiwan University, Taipei 10617, Taiwan; d03223108@ntu.edu.tw (C.-Y.C.); r03223121@ntu.edu.tw (C.-L.C.); d03223129@ntu.edu.tw (C.-M.W.)

**Keywords:** copper nanocluster, paper device, RGB analysis, antioxidative, thermoplastic lamination, sensor

## Abstract

Paper-based analytical devices are an emerging class of lightweight and simple-to-use analytical platform. However, challenges such as instrumental requirements and chemical reagents durability, represent a barrier for less-developed countries and markets. Herein, we report an advanced laminated device using red emitting copper nanocluster and RGB digital analysis for signal improvement. Upon RGB system assistance, the device signal-to-background ratio and the calibration sensitivity are highly enhanced under a filter-free setup. In addition, the calibration sensitivity, limit of detection, and coefficient of determination are on par with those determined by instrumental fluorescence analysis. Moreover, the limitation of using oxidation-susceptible fluorescent nanomaterials is overcome by the introduction of protecting tape barriers, antioxidative sheets, and lamination enclosing. The robustness of device is highly advanced, and the durability is prolonged to more than tenfold.

## 1. Introduction

Paper-based analytical devices have gained considerable attention due to several attractive attributes, such as low cost, lightweight, disposable, biocompatible, environment-friendly, and ease of modification [1,2,3,4]. Their inexpensiveness and simple-to-use properties offer underserved communities an opportunity to improve life quality in healthcare, environmental safety, and disease control [5,6,7]. However, remaining challenges, including robust quantitative and sensitive detection, elongated device durability, and straightforward manufacturing processes restrict the widespread usage of such devices [8,9]. Moreover, paper devices, in conjunction with fluorescence-based approaches that possess lower background and higher sensitivity than colorimetric methods, are still very limited. This is due to the requirement of not only indispensable excitation sources, but also suitable optical filter setups to cut off background fluorescence from original excitation, scattering light of paper substrates, and other presented fluorophores in sample matrix [5,6,7,10,11]. These limitations have restricted their use in less-developed countries and increased marketing barriers.

A good choice of optical sensing probe is a straightforward approach to solve the aforementioned device fabrication problems. Metal nanocluster (NC) (e.g., Au [12,13], Ag [14,15], and Cu [16,17]) is an emerging class of nanomaterial with large Stokes shifts fluorescence emission that can be used as sensors toward a wide range of targets, such as DNA/RNA [17,18,19], small biomolecules [20], metal ions [16,21], pH sensing [22,23,24], and cancer cells [25,26,27]. Due to sub-nanometer to 10 nm size range, their continuous electronic bands break into discrete energy levels, leading to dramatically different optical, electrical, and chemical properties, as compared to nanoparticles [28,29]. The molecule-like fluorescent properties are determined by size, capping ligands, and oxidation state of metals [30,31,32]. Their high photostability, large Stokes shifts, and good biocompatibility properties make them highly potent nanomaterials for applications in paper-based analytical devices [16,25,27]. For instance, filter paper strips embedded with Tb^3+^/AuNC applied to Hg^2+^ detection exhibited 500-fold higher sensitivity than that of the fluorescent dye-based approach [33,34]. A wax patterned plate-format paper-based device conjugated with size-tunable CuNC aggregates was also demonstrated to provide faster and more cost-effective H_2_S sensing than instrumental photoluminescence analysis [35]. However, the concerns of environment-induced harms on sensing reagents performance and device durability remains on these paper-based platforms.

In addition to probe picking, quantitative analysis with minimal instrumental requirements is another important task. Among digital analysis methods, RGB system is the most common mode [36,37,38,39,40], which represents colors through three tuples, red (R), green (G), and blue (B) [41,42]. The idea of three-color system was first reported by Jakob Christoffel Le Blon in 1722 [43]. The color reproduction can be adjusted by red, green, and blue lights, whose wavelengths are around 700 nm, 550 nm, and 440 nm, respectively [44]. Although the RGB color analysis is too simple to represent certain hues [45], it is still an easy and adequate approach to separate tints of images for quantitative analysis. The intensity scale of each monochrome is defined from 0 (dark) to 255 (bright). For example, blue color can be displayed by 255 of B value, while R and G values are 0. Similarly, red color represents 255 of R value, as its G and B values are 0. The RGB digital processing was therefore applied to colorimetric paper-based devices for convenient analysis [38,39,40]. 

In this study, the use of RGB system to assist a large Stokes shift nanomaterial-incorporated paper-based device for practical and promising quantitative analysis without requiring optical filters is investigated for the first time. A CuNC-incorporated antioxidative laminated paper device with RGB digital analysis is developed, where CuNC is selected as the model nanomaterial, due to its ease of oxidation, compared to Au and Ag [23,25,46]. The RGB digital analysis eliminates the matrix background without any complicated optical filter setup for convenient target detection. Owing to the red emitting color of CuNC, blue to green color regions of fluorescence interference can be easily removed by image conversion into R tuple. Not only the calibration sensitivity, but also the coefficient of determination is highly improved under this filter-free setup by observing the R tuple only. The design, moreover, integrates lamination strategy and antioxidative sheets to protect oxidation-susceptible CuNC, prolonging the device shelf life to more than tenfold.

## 2. Results and Discussion

**Figure 1:**
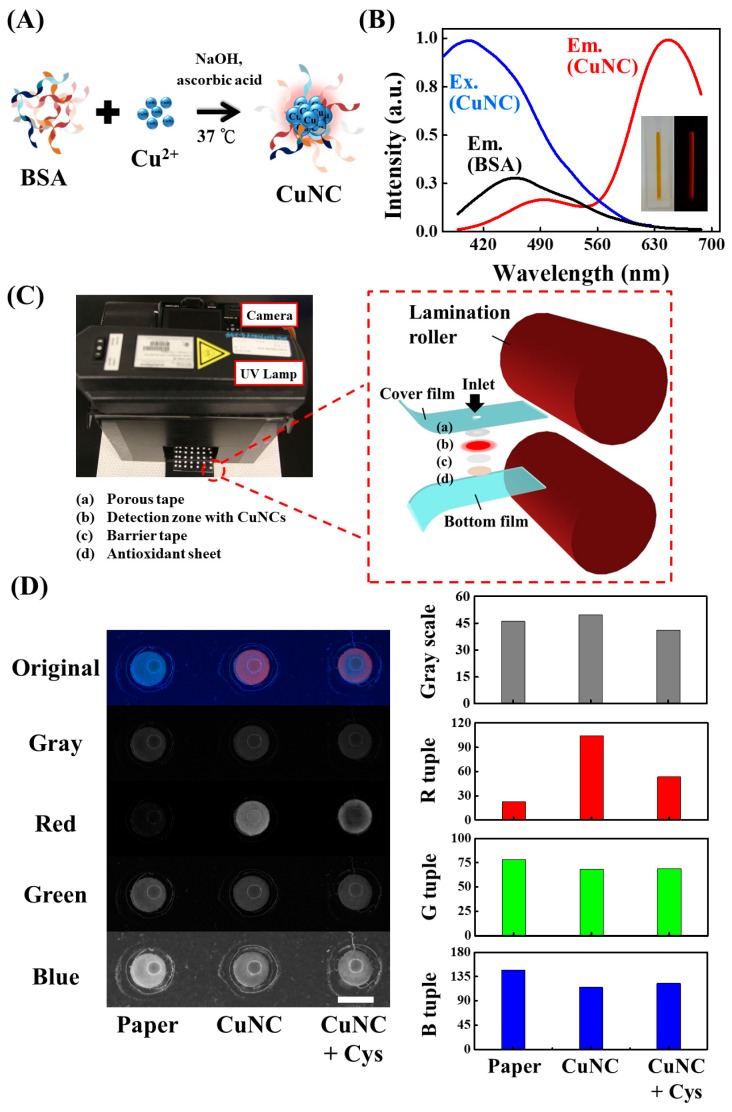
(**A**) Schematic illustration of the CuNC probe synthesis. (**B**) Overlapping excitation and emission spectra of CuNC and emission spectrum of BSA solution. Inset: photographs of the CuNC solution under visible light (left) and 365 nm UV illumination (right). (**C**) The device operation setup (left) and schematic illustration of laminated CuNC–paper composite sensing device (right). (**D**) Photograph analysis of different laminated paper substrates including raw paper material, CuNC–paper composite, and CuNC–paper composite with cysteine under different color tuples. The photo image (left) is converted into numerical histograms (right), representing their corresponding intensity values at gray scale, red (R), green (G), and blue (B) tuples. The scale bar is 5 mm.

A red-emitting CuNC synthesis method using commercially available bovine serum albumin (BSA) under ascorbic acid-assisted reduction is employed in this study, as displayed in Figure 1A. BSA provides not only steric protection via the strong Cu–S bonding during the encapsulation process [22], but also performs as a reductant when the reaction pH is adjusted to ~12 [12]. After 30 min of BSA and copper ion solution incubation, ascorbic acid is introduced into the mixture as an assisting reductant for rapid CuNC formation. As shown in inset of Figure 1B, the prepared solution is pale brown with red fluorescence emission under 365 nm of excitation, suggesting the formation of CuNC. The distinct UV–vis absorption peak of CuNC at ~350 nm (Figure S1, Supplementary Materials) indicates molecule-like optical transition attributed to quantum confinement effect [47,48], which is significantly different from localized surface plasmon resonance of copper nanoparticles [49,50]. The fluorescence property (Figure 1B) of synthesized CuNC displays maximum excitation and emission peaks at 392 and 648 nm, respectively, with a large Stokes shift of 256 nm. To characterize the synthesized 3 nm nanocluster, fluorescence lifetime, XPS, HRTEM, DLS (Figures S2–S5, Supplementary Materials) analysis were carried out, and the results are carefully discussed in the Supplementary Materials. The capability of synthesized CuNC as a sensing probe is demonstrated by detecting cysteine, a critical disease indicator, via the strong interaction between them [51,52,53,54]. As shown in Figure S6 (Supplementary Materials), fluorescence spectra reveal that cysteine addition lowers the fluorescence emission intensity of CuNC solution. Meanwhile, a slight blue shift in the emission spectra is also observed resulting from size change of CuNC. The observations of different thiol group-containing small molecules test and DLS characterization (Figures S5, S7, and S8, Supplementary Materials) suggest that the cysteine-induced CuNC fluorescence quenching is attributed to the decomposition of CuNC into smaller clusters or metal complexes, as depicted in Figure S9 (Supplementary Materials). (Detailed mechanism discussions are illustrated in the Supplementary Materials).

To build a fluorescent nanomaterial-based sensing device, the problem of oxidation-induced material property change needs to be solved. A lamination strategy is proposed to overcome this restriction and prolong device lifetime. As illustrated in Figure 1C, the device enclosed within thermoplastic films consists of a detection zone containing CuNC, an antioxidant sheet (loaded with ascorbic acid), and two protecting tapes. The analytical data of the fabricated device is collected as photographs recorded with a digital camera (Leica D-LUX 6, Leica Camera AG, Wetzlar, Germany) as shown in Figure 1D. Under UV lamp (6 W, 365 nm) illumination, the CuNC–paper composite shows clear red color emission, whereas the raw paper material does not. After cysteine introduction, this red emission reduces, due to cysteine-induced metal cluster etching. As can be seen in Figure 1D, the blue color background in the original image complicates the visualization detection and interferes with following quantitative analysis. The use of previously-studied gray scale conversion [55,56,57], however, still gives independent assay brightness reading regardless of CuNC existence. This indicates that the background interference stands unresolved when only gray scale analysis is employed without filter setups. On the other hand, photograph colors can be separated into R, G, and B tuples under the RGB system analysis. In Figure 1D, similar intensities in G and B tuples, regardless of cysteine addition, indicate severe background interference, because of scattering and reflection of excitation light and matrix auto-fluorescence, which mostly appear in the blue-green region. By contrast, these background noise levels are drastically lower under R tuple with the existence of CuNC, which emit red color due to the large Stokes shift of over 250 nm. Moreover, the image contrast change of CuNC–paper composites under cysteine addition is highly enhanced comparing to the original one without any digital analysis processing. Since the signal-to-background (S/B) ratio is defined as the intensity ratio of CuNC–paper composite over raw paper [15,58], the S/B ratio in R tuple is enhanced over 4 times as compared to other color tuples. This RGB analysis is therefore a useful tool to greatly reduce the fluorescence background of laminated paper device without requiring any optical filter setup.

**Figure 2:**
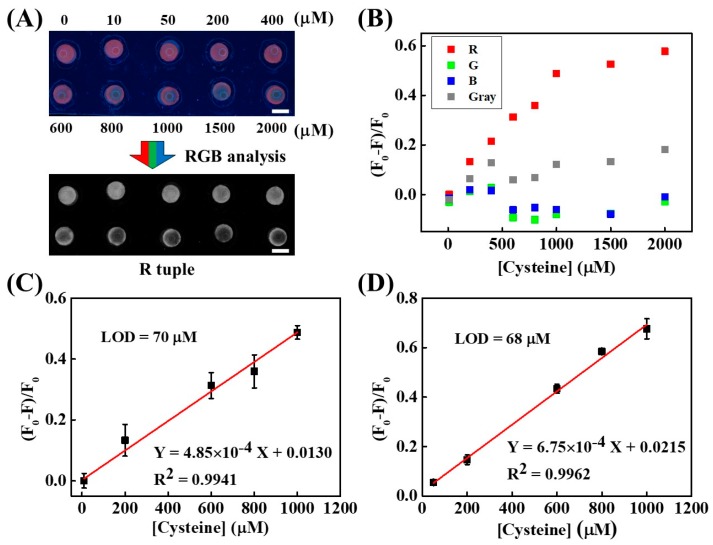
(**A**) Photographs of a laminated CuNC–paper composite device under different cysteine concentration treatments. Upper image: the device under 365 nm UV illumination. Lower image: the image after conversion into the R tuple. The scale bars are 5 mm. (**B**) The correlation in between the device image intensity and cysteine concentration, where *F*_0_ and *F* respectively represent the value in the absence and presence of cysteine. (**C**) The calibration curve derived from the R tuple analysis. (**D**) The calibration curve of cysteine detection by a fluorescence spectrometer (SpectraMax^®^ i3x Multi-Mode microplate reader, Molecular Devices, San Jose, CA, USA). Each data point is obtained from average value of three samples (*N* = 3). The error bars indicate the standard deviation.

The capability of CuNC-based paper device fluorescence emission in R tuple is further tested by different concentrations of cysteine ranging from 0 to 2000 µM, as presented in Figure 2A. The red emission of CuNC–paper composite reduces when cysteine concentration is raised. After R tuple analysis, the contrast difference is highly improved especially at lower concentrations. It is found that the intensity of R value dramatically decreases along with increasing cysteine concentration, whereas the G and B values exhibit no obvious change as shown in Figure 2B. It is important to mention that the calibration sensitivity (the slope of calibration curve) of R value changes is over 4-fold higher than others as displayed in Figure S10 (Supplementary Materials). In addition, the coefficient of determination (*R*^2^) in R tuple is higher than 0.99, while others are lower than 0.50. The calibration curve is constructed from the intensity value in R tuple of the RGB system, and represents a linear response within the 10–1000 µM cysteine range (Figure 2C). Based on the limit of detection (LOD) definition as the standard deviation of y-intercept to the slope of calibration curve ratio over 3 [59], the LOD is calculated to be 70 μM. The cysteine quantification by fluorescence spectrometer is also carried out, as shown in Figure 2D. The calibration sensitivity, coefficient of determination, and LOD of this device are on par with those obtained from instrumental fluorescence analysis. These results indicate that the RGB system can improve quantitative analysis and minimize the instrumental requirements in the optical filter-free paper device using red-emitting nanomaterials as sensing probes.

**Figure 3:**
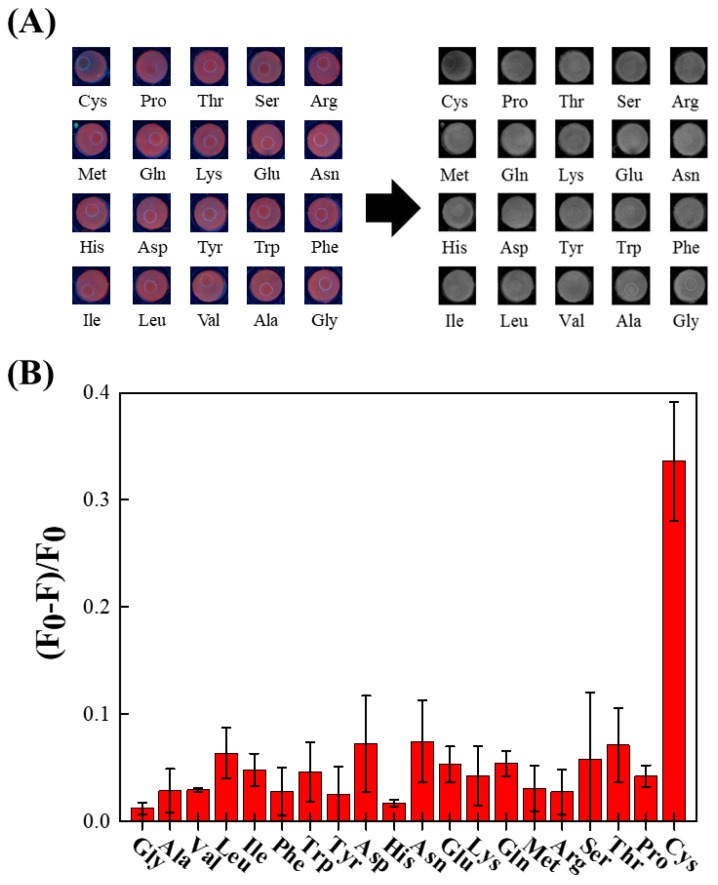
(**A**) Photographs representing device responses to various amino acids. All the tested amino acids are prepared as 500 μM aqueous solutions. Left: the device under 365 nm UV illumination. Right: the image after conversion into the R tuple. (**B**) The device fluorescence responses in R tuple under various amino acid treatments.

**Figure 4:**
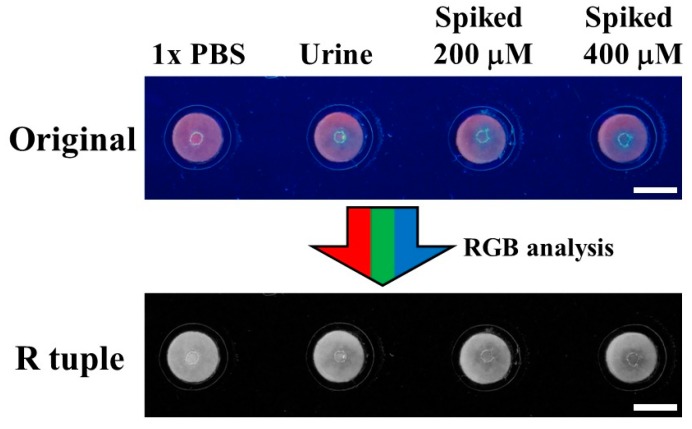
Photographs of a laminated CuNC–paper composite device under different treatments. From left to right: PBS buffer, urine, urine spiked with 200 μM cysteine, urine spiked with 400 μM cysteine. Upper image: the device under 365 nm UV illumination. Lower image: the image after conversion into the R tuple. The scale bars are 5 mm.

The fabricated devices also present excellent selectivity toward cysteine over other natural amino acids, as shown Figure 3. To test the proposed device for real samples, the direct determination of cysteine in complex matrixes such as urine is studied. The physiological level of cysteine in urine can mirror its content in plasma, while this non-invasive detection has several advantages, including patient compliance, easy sample handling, and convenient collection [60]. As shown in Figure 4, the fresh urine sample donated by the healthy volunteer is introduced onto the CuNC-based paper device without any pretreatment. The detected average urinary cysteine concentration is calculated to be 130.90 ± 9.18 μM (Table 1), which is in good agreement with the reported results [61,62]. The recovery of cysteine by standard addition in urine samples ranges from 102% to 104% with *a* < 10% relative error, indicating the high accuracy and acceptable reproducibility of our device.

Using environment sensitive materials as sensing probes in paper-based devices may result in reactivity loss, due to oxidative degradation with exposed reagents. To conquer this hindrance, the lamination strategy can be utilized to improve the reagent durability, as well as device mechanical properties. Compared to recently reported laminated paper devices [63,64], two main significant advances are presented in this study: (1) tape barriers, and (2) antioxidative sheets. 

**Figure 5:**
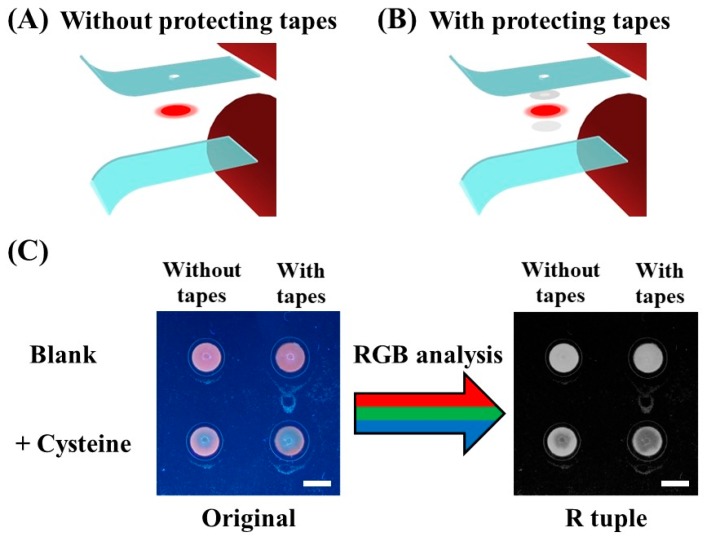
Schematic illustrations of the lamination process without (**A**) and with (**B**) tape barriers. (**C**) Photographs of the laminated CuNC–paper composite device without/with the use of protecting tapes for cysteine detection (1 mM). Left image: photo image under 365 nm UV illumination. Right image: the same photo image after conversion into the R tuple. The scale bars are 5 mm.

The introduced tape layers protect reagents in the detection area from lamination material interferences. As illustrated in Figure 1C and Figure 5B, the tape above the detection zone is employed to avoid the cover film adhesive contamination, while the other one is a barrier to prevent the antioxidant sheet from direct contact. Furthermore, these layers also highly reduce the thermoplastic adhesives’ permeation into paper substrates, preventing reaction inhomogeneity. As shown in Figure 5, the analyte solution is difficult to wick through paper substrates, and react with CuNC homogeneously without the use of tape barriers. 

**Figure 6:**
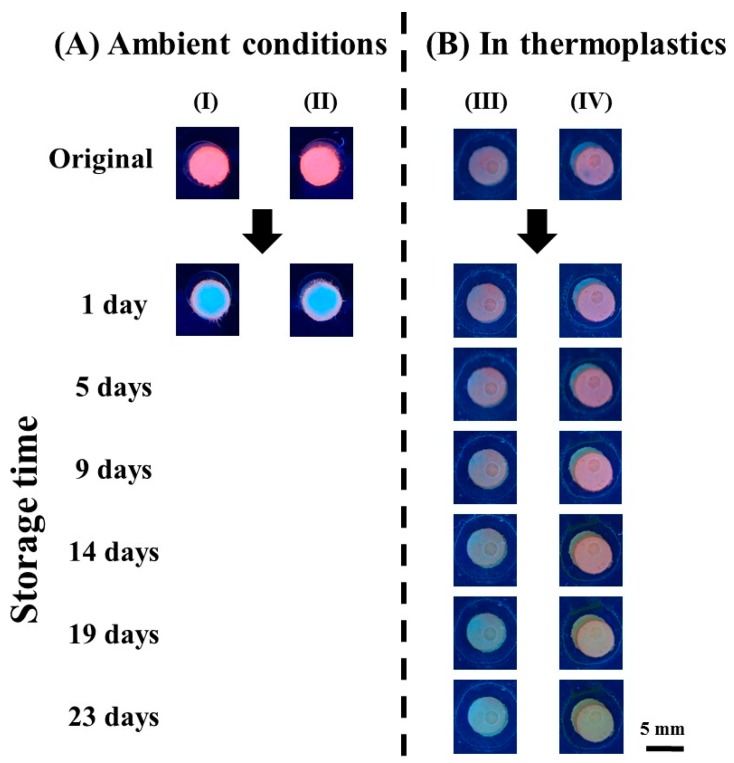
(**A**) CuNC–paper composites were placed under ambient condition for one day without (I) and with (II) the use of antioxidative sheets. (**B**) CuNC–paper composites were enclosed in thermoplastics without (III) and with (IV) the use of antioxidative sheets. The scale bars are 5 mm.

The advantage of antioxidative sheets insertion in laminated platforms is demonstrated in Figure 6. The red fluorescence emission of CuNC–paper composites without lamination protection (column I) and with antioxidative sheets (column II) disappear after just one day of ambient storage. This fast degradation can be attributed to CuNC oxidation, owing to their low oxidation potential (*E*^0^ = 0.34 V) [25]. On the other hand, the protection provided by thermoplastic films can extend CuNC lifetime, as displayed in Figure 6B. Without antioxidative sheets (column III), the CuNC–paper composites keep their fluorescence emission property only up to 5 days via laminated PET film protection. The low oxygen transmission rate of PET films (8.4 cm^3^ m^−2^ day^−1^ atm^−1^) [65] effectively slows down the cluster degradation, but cannot prevent air penetration completely. Inspired by common food packaging techniques, antioxidative sheets are placed underneath the CuNC–paper composites, and enclosed by thermoplastic films altogether, to greatly prolong the device shelf life more than 20 days, as displayed in column IV of Figure 6B. This suggests that thermoplastic films can lower the influence from air transmission, while the antioxidative reagent is able to devour the penetrated oxygen from atmosphere. Although the desired durability of disposable analytical devices depends on the reagents used [63,66,67], we achieved significant improvement as the shelf life of easily oxidized model nanomaterial CuNC is extended to more than tenfold. It should be noted that this antioxidation strategy is also promising for other devices via simple operation and requirements, without any substrate chemical modification. In addition, the effect of varied temperatures and humidity levels on detected results are expected to be minimal, due to protection from the antioxidative sheets and polymer films. Thus, upon the combination of thermoplastic films, protecting tape barriers, and antioxidative sheets, the use of oxidation-susceptible nanomaterials in RGB analysis-assisted paper device is achieved.

## 3. Experimental Section

**Materials**. Whatman filter paper (No. 40) with 8 µm pores was selected as the platform material. Thermal bonding lamination films (polyethylene terephthalate–ethylene-vinyl acetate, PET–EVA) with 80 µm thickness were purchased from Taiwan Halee international Co., Ltd. (Taipei, Taiwan). Bovine serum albumin (BSA) was provided by Bioman Scientific (Taipei, Taiwan). Twenty natural amino acids, l-glutathione, 3-mercaptopropionic acid, cysteamine, copper(II) nitrate (Cu(NO_3_)_2_), ascorbic acid, and chitosan were purchased from Sigma-Aldrich (St. Louis, MO, USA). Sodium hydroxide (NaOH), sodium dihydrogen phosphate, and sodium hydrogen phosphate were obtained from SHOWA (Tokyo, Japan). Ultrapure water (>18.2 MΩ cm), generated from ELGA PURELAB classic system (Taipei, Taiwan), was used in all experiments.

**Synthesis of Red-Emitting Fluorescent CuNC**. BSA solution (2.5 mL of 40 mg/mL) was first mixed with 2.5 mL Cu(NO_3_)_2_ solution (10 mM), and stirred for 5 min at 37 °C. Five hundred microliters of 1 M NaOH solution was then introduced to adjust the solution pH to 12, changing the color of the solution from green to violet. After 30 min, 1 mL freshly prepared ascorbic acid solution (0.5 M) was slowly dropped into the mixture under vigorous stirring. After 2 h of incubation, the solution color changed from violet to pale brown, which suggested the formation of CuNC. This CuNC solution was stored at 4 °C in the dark before use.

**Characterization of CuNC**. Absorption and fluorescence spectra of CuNC solutions were measured by the Thermo Evolution UV-220 spectrophotometer (Thermo Fisher Scientific Inc., Waltham, MA, USA) and the Varian Cary Eclipse fluorescence spectrophotometer (Varian Inc., Palo Alto, CA, USA), respectively. Dynamic light scattering (DLS) measurements were recorded by the Malvern Zetasizer Nano S Instrument (Malvern Instruments Ltd., Malvern, Worcs, UK) and all the scattered photons were collected at the scattering angle of 173°. High-resolution transmission electron microscopy (HRTEM) experiments were performed on the Philips Tecnai F20 G2 field-emission high-resolution transmission electron microscope (Thermo Fisher Scientific Inc., Waltham, MA, USA) with the use of 200 kV electron beams. X-ray photoelectron spectra (XPS) were collected by the Kratos Axis Ultra DLD X-ray photoelectron spectrometer microprobe (Kratos Analytical Ltd., Stretford, Manchester, UK) with a mono Al K_α_ radiation source (1486.71 eV).

**Fabrication of Laminated CuNC–Paper Composite Devices**. As shown in Figure 1C, each paper-based sensing strip enclosed within thermoplastic films consists of a detection zone with CuNC, an antioxidant sheet, and two protecting tapes. Detection zones were prepared by introducing 6.0 µL of CuNC solution onto a 5 mm diameter circular paper piece shaped by a commercial hole puncher, and vacuum dried in a desiccator for 20 min. The antioxidant sheet was fabricated by immersing a circular paper piece into the 0.5 M ascorbic acid/chitosan hydrogel for 4 min soaking. The porous tape (fabricated by a hole puncher) above the detection zone was employed to avoid the cover film adhesive contamination, while the other one was a barrier to prevent the antioxidant sheet direct contact. Before lamination, a 2 mm hole was drilled on the cover thermoplastic film for detection zone sample introduction. Finally, all components from top to down: cover film with the inlet hole, porous tape, detection zone loaded with CuNC, barrier tape, antioxidant sheet, and bottom film, were aligned and enclosed together through a heated roll laminator (TCC-6000, Tah Hsin Industrial Corp., Taichung, Taiwan). The lamination process was controlled under a constant rolling speed of 3 cm/s at 130 °C. The multi-layer components were therefore enclosed inside the thermoplastic films when heated through the roll laminator. An extra layer of thermoplastic film was adhered on top of the device to protect the detection zone from air exposure for storage purpose. Due to less adhesive of a single layer thermoplastic film, this additional layer can be easily peeled off from the device at the time of usage.

**Analytical Assays with a Digital Camera**. During target analysis, 6.0 µL of sample was introduced through the inlet, and the solution transited to the detection zones by capillary wicking. After 5 min of reaction, the CuNC emission color change was visualized under handheld 365 nm UV lamp (6 W) illumination from a UVP UVGL-58 lamp (Analytik Jena US, Upland, CA, USA). The device was placed in the dark to minimize ambient light influences, and the photo images were captured with a Leica D-LUX 6 digital camera (Leica Camera AG, Wetzlar, Germany). The collected images were analyzed by Adobe Photoshop CC 2014 (Adobe Systems Incorporated, San Jose, CA, USA), which separated the color into RGB tuples, or in gray value. The value in R tuple was selected for analyte quantification, due to its high S/B ratio and great coefficient of determination.

## 4. Conclusions

In conclusion, a laminated paper-based device using oxidation-susceptible CuNC for filter-free fluorescence detection of cysteine with RGB system-assisted signal improvement is reported. With the assistance of the RGB analysis, the S/B ratio and calibration sensitivity of CuNC-based paper device are enhanced for more than 4 times without using any optical filter, while the coefficient of determination stays above 0.99, suggesting great potential for quantitative analysis. The challenges of using environment sensitive materials are also overcome with the incorporation of protecting tape barriers, antioxidative sheets, and lamination enclosing design, which greatly improves the device durability to more than tenfold. The integrated nanomaterial-based sensor design, device lamination process, and RGB system-assisted signal improvement have further expanded the opportunity of paper sensing devices with lowered instrumental requirements. We envision this design to be potent in integrating with common portable devices, such as cellular phones, for real time and on-site diagnosis applications.

## Figures and Tables

**Table 1:** Determination of cysteine in urine samples.

Spiked (μM)	Found *^a^* (μM)	Recovery *^a^* (%)
0	130.90 ± 9.18	-
200	204.71 ± 11.18	102.36 ± 5.59
400	416.21 ± 39.17	104.05 ± 9.79

*^a^* Average of three determinations ± standard deviation.

## Supplementary Materials

The following are available online at http://www.mdpi.com/2079-4991/8/2/97/s1, Figure S1: UV–Vis absorption spectra, Figure S2: Fluorescence decay profile, Figure S3: X-ray photoelectron spectra, Figure S4: HRTEM image, Figure S5: DLS spectra, Figure S6: Fluorescence emission spectra of CuNC mixed with cysteine, Figure S7: Fluorescence emission spectra of CuNC mixed with different thiol group-containing molecules, Figure S8: DLS spectra of CuNC mixed with different thiol group-containing molecules, Figure S9: Schematic illustration of the cysteine detection mechanism, Figure S10: The correlation in between cysteine concentration and device image intensity in different digital analysis system.
