# Supplementary material for: Laminated Copper Nanocluster Incorporated Antioxidative Paper Device with RGB System-Assisted Signal Improvement
Source: Nanomaterials (Basel). 2018 Feb 9;8(2):97. doi: 10.3390/nano8020097 (PMC5853728; doi:10.3390/nano8020097)
Supplement: Supplementary file 1 [file nanomaterials-08-00097-s001.pdf]

Supporting Information

Laminated Copper Nanocluster Incorporated Antioxidative Paper Device with RGB System-Assisted Signal Improvement

Chong-You Chen‡, Chia-Lin Chen‡, Chang-Ming Wang, and Wei-Ssu Liao*

Department of Chemistry, National Taiwan University, Taipei 10617, Taiwan

E-mail: wsliaochem@ntu.edu.tw

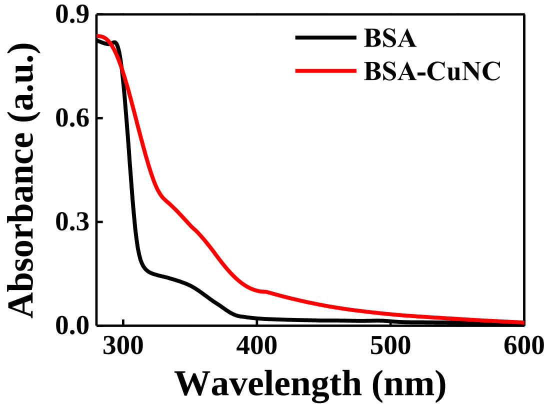

Figure S1. UV-Vis absorption spectra of BSA-CuNC and BSA solutions.

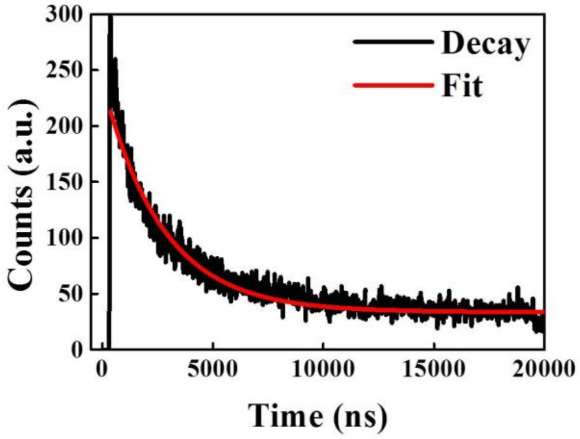

Figure S2. Fluorescence decay profile (λ_{ex} = 350 nm and λ_{em} = 650 nm) of CuNC obtained using a time-correlated single photon counting technique. The measured fluorescence lifetime of CuNC is 2.7 μs.

The long radiative lifetime of 2.7 μs in the microsecond range is a result of efficient intersystem crossings between the singlet and the triplet states,[1] which is similar to characteristics of luminescent BSA-protected 25-atom gold nanoclusters that display ligand-metal charge transfer or metal-metal interactions.[2]

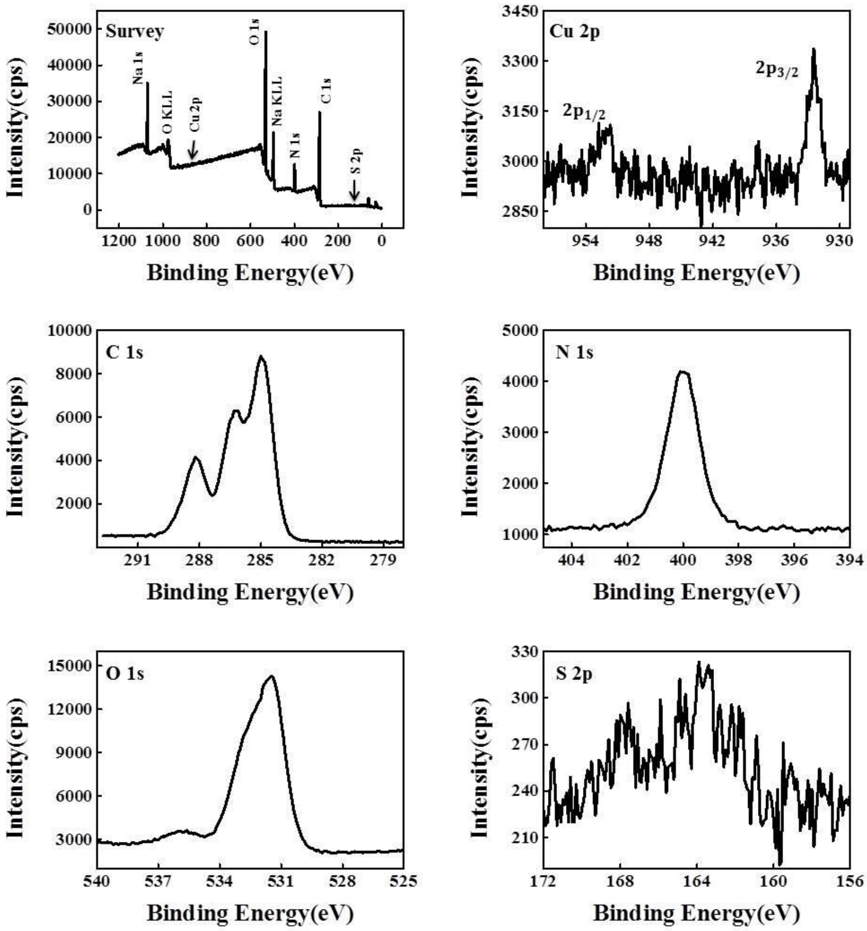

Figure S3. X-ray photoelectron spectra of the elements (Cu 2p, C 1s, N 1s, O 1s, S 2p) present in the CuNC sample.

The analysis shows two prominent peaks of Cu^0 at 932.5 eV and 952.1 eV, which are assigned to be Cu $2p_{3/2}$ and Cu $2p_{1/2}$. The lack of Cu^{2+} peak at 943 eV indicates that the as-prepared CuNC does not contain Cu^{2+}. However, due to only 0.1 eV difference between Cu^+ and Cu^0, the oxidation state of Cu in prepared CuNC samples most likely lies in between 0 and +1. Furthermore, other matrix elements are also found in the survey and single element spectra.

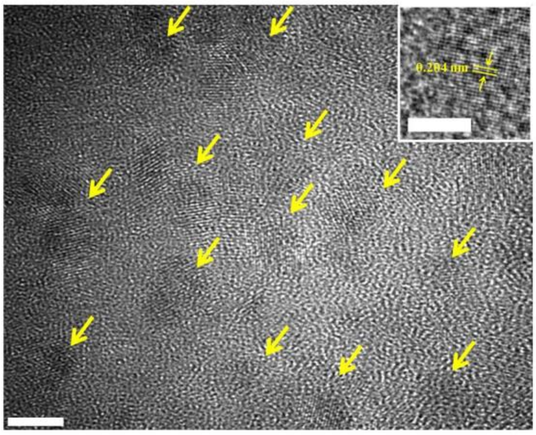

Figure S4. HRTEM image of the prepared CuNC. The scale bars are 5 nm.

The prepared CuNC retain crystalline structures and the metal cores are smaller than 5 nm. The inset image shows the crystalline particle with a lattice spacing of 0.204 nm, corresponding to the (111) plane of Cu.

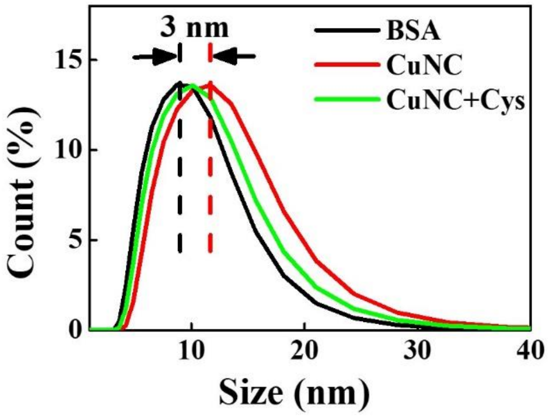

Figure S5. DLS spectra of BSA, CuNC, and CuNC with 2.5 mM cysteine solutions.

The size of CuNC was determined via dynamic light scattering (DLS) measurements that reveals a hydrodynamic size distribution of ~12 nm, larger than the ~9 nm BSA templates. It should be noted that this 12 nm distribution in DLS spectrum is not contributed by larger copper nanoparticles due to the absence of LSPR absorption peak (Fig. S1)

and the lack of particles larger than 5 nm in HRTEM images (Fig. S4). The 3 nm difference in size distribution between BSA and CuNC is therefore determined to be the metal cluster core size.

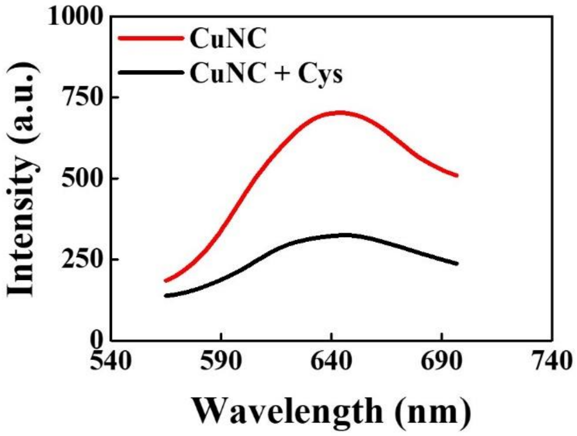

Figure S6. Fluorescence emission spectra of CuNC in the absence and presence of 2 mM cysteine.

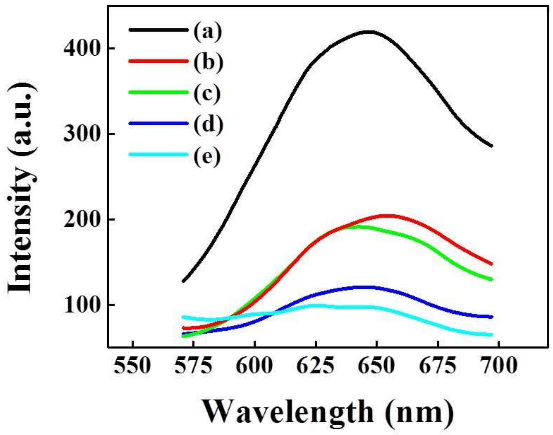

Figure S7. Fluorescence emission spectra of CuNC (a) when mixed with 2.5 mM different types of thiol group-containing molecules: cysteine (b), cysteamine (c), 3-mercaptopropionic acid (d), and glutathione (e).

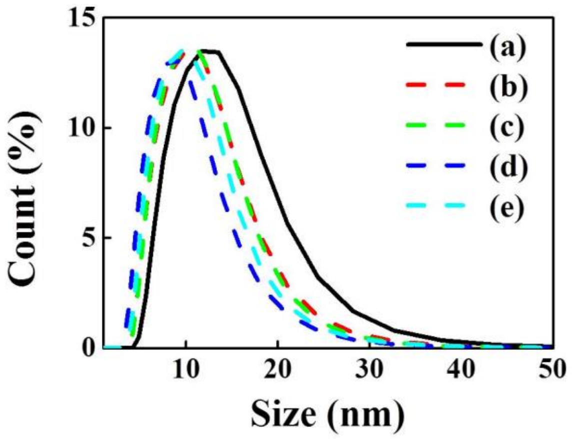

Figure S8. DLS spectra of CuNC (a) when mixed with 2.5 mM different types of thiol group-containing molecules: cysteine (b), cysteamine (c), 3-mercaptopropionic acid (d), and glutathione (e).

Due to thiol's strong affinity toward copper,[3] two possible sources could contribute to this fluorescence quenching phenomenon. First, cysteine could penetrate into the BSA molecule capped clusters to compete with ligands on the metal surface, leading to ligand replacement and cluster destabilization, and finally resulting in cluster aggregation and fluorescence quenching. On the other hand, the cysteine thiol groups could decompose copper nanoclusters into smaller clusters or metal complexes, and resulting in decreased fluorescence emission intensity as well as peak shifts. The results of DLS measurements as presented in Fig. S5 show that the size distribution of Cys-BSA-CuNCs is smaller than BSA-CuNCs, and no larger particle distribution is found. These observations rule out the first perspective ligand replacement induced quenching mechanism. To further investigate the other thiol induced etching process, different small ligand molecules containing thiol group, such as cysteamine, 3-mercaptopropionic acid, and glutathione are tested, as

shown in Fig. S7-S8. It is found that similar fluorescence emission quenching as well as cluster size shrinking are also observed with the use of these molecules. The similar fluorescence reduction trend and size shrinking tendency reveal a common factor governing these observations. These results lead us to believe that the cysteine-induced copper nanoclusters fluorescence emission quenching is attributed to the thiol etching process as illustrated in Fig. S9.[4]

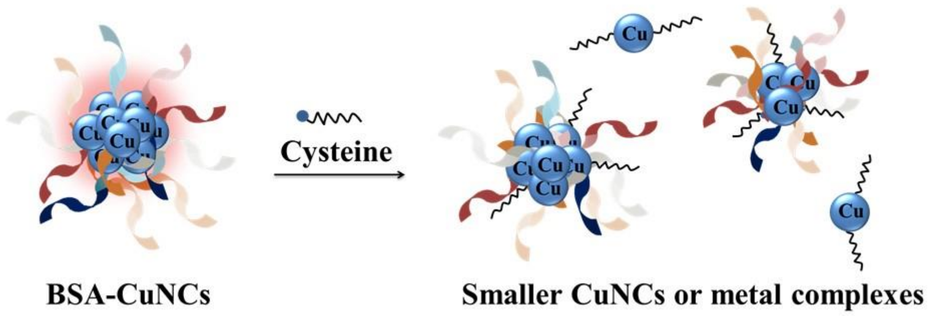

Figure S9. Schematic illustration of the cysteine detection mechanism.

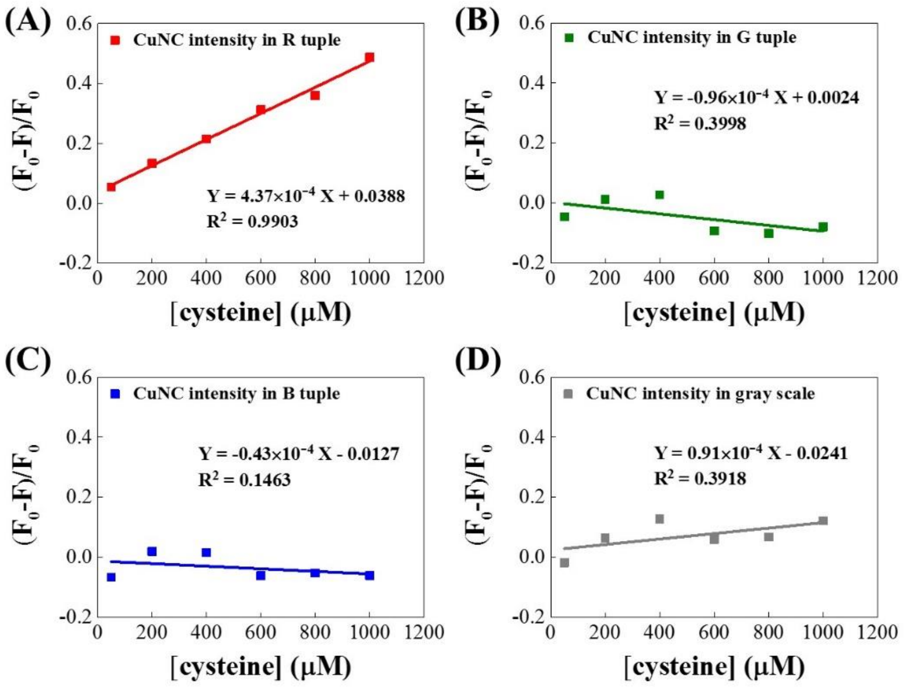

Figure S10. The correlation in between cysteine concentration and device image intensity in R tuple (A), G tuple (B), B tuple (C), and gray scale (D), where F_0 and F respectively represent the value in the absence and presence of cysteine.

References

(1) Jia, X. F.; Li, J.; Han, L.; Ren, J. T.; Yang, X.; Wang, E. K., DNA-Hosted Copper Nanoclusters for Fluorescent Identification of Single Nucleotide Polymorphisms. *ACS Nano* **2012**, *6*, 3311-3317.

(2) Wen, X. M.; Yu, P.; Toh, Y. R.; Hsu, A. C.; Lee, Y. C.; Tang, J., Fluorescence Dynamics in BSA-Protected Au_{25} Nanoclusters. *J. Phys. Chem. C* **2012**, *116*, 19032-19038.

(3) Kroneck, P. M. H.; Vortisch, V.; Hemmerich, P., Model Studies on The Coordination of Copper in Biological systems. The Deprotonated Peptide Nitrogen as a Potential Binding Site for Copper(II). *Eur. J. Biochem.* **1980**, *109*, 603-612.

(4) Yuan, X.; Tay, Y.; Dou, X.; Luo, Z.; Leong, D. T.; Xie, J., Glutathione-Protected Silver Nanoclusters as Cysteine-Selective Fluorometric and Colorimetric Probe. *Anal. Chem.* **2013**, *85*, 1913-1919.
